# Supplementary material for: Association between Breast Cancer and Second Primary Lung Cancer among the Female Population in Taiwan: A Nationwide Population-Based Cohort Study
Source: Cancers (Basel). 2022 Jun 16;14(12):2977. doi: 10.3390/cancers14122977 (PMC9221143; doi:10.3390/cancers14122977)
Supplement: Supplementary file 1 [file cancers-14-02977-s001.zip › cancers-1749266-supplementary.pdf]

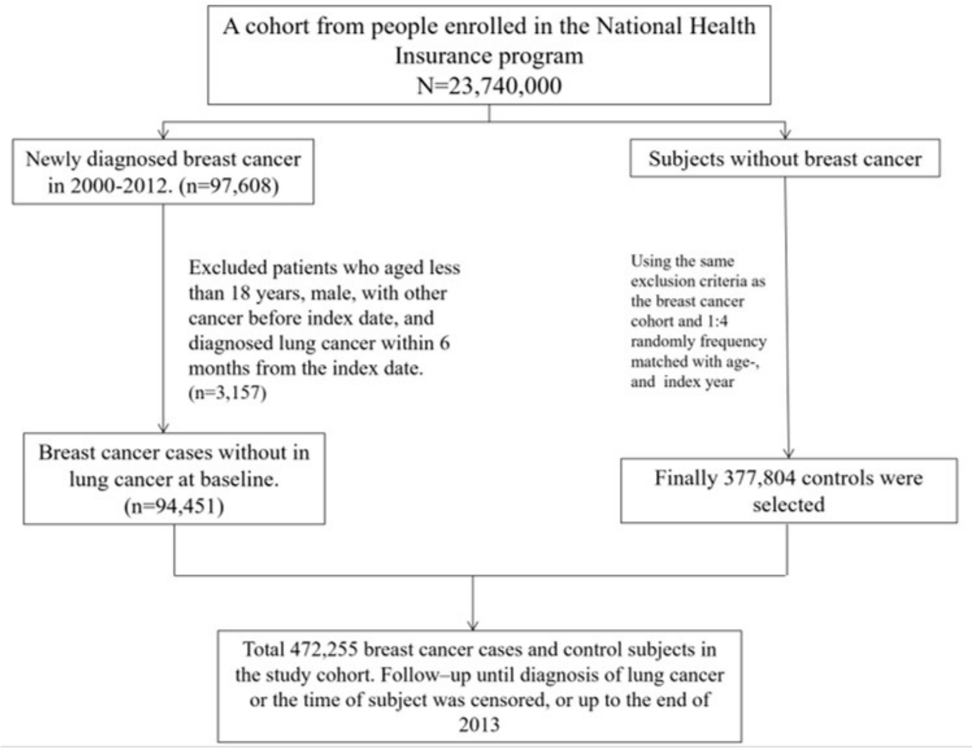

Figure S1. Study flowchart.

Table S1. The standardized incidence ratio (SIRs) and 95% confidence interval for lung cancer in breast cancer.

Malignancy (ICD-9-CM)	Breast Cancer		
	**Observed Number**	**Expected Number**	**SIR (95% CI)**
Lung cancer (162)	447	146.2	3.06(2.79, 3.36) ***
SIR: standard incidence density rate			

*** $p < 0.001$.
